# Mechanisms of Formation, Structure, and Dynamics of Lipoprotein Discs Stabilized by Amphiphilic Copolymers: A Comprehensive Review

**DOI:** 10.3390/nano12030361

**Published:** 2022-01-23

**Authors:** Philipp S. Orekhov, Marine E. Bozdaganyan, Natalia Voskoboynikova, Armen Y. Mulkidjanian, Maria G. Karlova, Anna Yudenko, Alina Remeeva, Yury L. Ryzhykau, Ivan Gushchin, Valentin I. Gordeliy, Olga S. Sokolova, Heinz-Jürgen Steinhoff, Mikhail P. Kirpichnikov, Konstantin V. Shaitan

**Affiliations:** 1 Faculty of Biology, Lomonosov Moscow State University, 119991 Moscow, Russia; bozdaganyan@mail.bio.msu.ru (M.E.B.); tayojniy.drug@gmail.com (M.G.K.); sokolova@mail.bio.msu.ru (O.S.S.); kirpichnikov@inbox.ru (M.P.K.); 2 Faculty of Biology, Shenzhen MSU-BIT University, Shenzhen 518172, China; 3 Institute of Personalized Medicine, Sechenov University, 119146 Moscow, Russia; 4 N.N. Semenov Federal Research Center for Chemical Physics, Russian Academy of Sciences, 119991 Moscow, Russia; 5 Department of Physics, University of Osnabrück, Barbarastrasse 7, 49076 Osnabrück, Germany; natalia.voskoboynikova@uni-osnabrueck.de (N.V.); armen.mulkidjanian@uni-osnabrueck.de (A.Y.M.); hsteinho@uni-osnabrueck.de (H.-J.S.); 6 Faculty of Bioengineering and Bioinformatics and Belozersky Institute of Physico-Chemical Biology, Lomonosov Moscow State University, 119234 Moscow, Russia; 7 Research Center for Molecular Mechanisms of Aging and Age-Related Diseases, Moscow Institute of Physics and Technology, 141700 Dolgoprudny, Russia; yudenkoan@gmail.com (A.Y.); alina.remeeva@phystech.edu (A.R.); rizhikov@phystech.edu (Y.L.R.); ivan.gushchin@phystech.edu (I.G.); valentin.gordeliy@ibs.fr (V.I.G.); 8 Institute of Biological Information Processing (IBI-7: Structural Biochemistry), Forschungszentrum Jülich, 52428 Jülich, Germany; 9 Institut de Biologie Structurale J.-P. Ebel, Université Grenoble Alpes-CEA-CNRS, 38000 Grenoble, France; 10 JuStruct: Jülich Center for Structural Biology, Forschungszentrum Jülich, 52428 Jülich, Germany; 11 Shemyakin-Ovchinnikov Institute of Bioorganic Chemistry, Russian Academy of Sciences, 117997 Moscow, Russia

**Keywords:** SMA, DIBMA, amphiphilic copolymers, lipodiscs, nanolipoparticles, structural biology

## Abstract

Amphiphilic copolymers consisting of alternating hydrophilic and hydrophobic units account for a major recent methodical breakthrough in the investigations of membrane proteins. Styrene–maleic acid (SMA), diisobutylene–maleic acid (DIBMA), and related copolymers have been shown to extract membrane proteins directly from lipid membranes without the need for classical detergents. Within the particular experimental setup, they form disc-shaped nanoparticles with a narrow size distribution, which serve as a suitable platform for diverse kinds of spectroscopy and other biophysical techniques that require relatively small, homogeneous, water-soluble particles of separate membrane proteins in their native lipid environment. In recent years, copolymer-encased nanolipoparticles have been proven as suitable protein carriers for various structural biology applications, including cryo-electron microscopy (cryo-EM), small-angle scattering, and conventional and single-molecule X-ray diffraction experiments. Here, we review the current understanding of how such nanolipoparticles are formed and organized at the molecular level with an emphasis on their chemical diversity and factors affecting their size and solubilization efficiency.

## 1. Introduction

The study of three-dimensional structures of integral membrane proteins (MPs) is one of the main tasks of molecular biology. Under natural conditions, MPs are embedded in biomembranes of complex composition mutually influencing each other [1,2]. For detailed structural and functional studies, MPs need to be isolated from this environment and purified while maintaining their stability and activity, which is a much more laborious task than the isolation and purification of soluble proteins. Therefore, much effort has been focused on new methodologies to improve the solubilization and stabilization of MPs [3].

Over the last few decades, a number of techniques have been developed addressing this problem, including the use of amphipols [4,5], bicelles [6], and membrane-scaffolding proteins (MSPs) [7]. However, the necessity for preliminary solubilization by conventional detergents does not preserve native lipids surrounding MPs and represents a major drawback of these methodologies.

A new promising approach is the use of amphiphilic copolymers to solubilize MPs directly in their natural environment in the form of polymer-bound lipoprotein nanoparticles, hereafter termed lipodiscs, although multiple synonymous and related terms exist, including lipodisqs [3], lipodisks [8], lipid nanodisks [9] (not to be confused with nanodiscs prepared using MSPs), maleic acid copolymer particles (MACPs), and SMALPs/DIBMALPs [10] (for lipoprotein particles formed by the two most popular copolymers, SMA and DIBMA, see below). Amphiphilic copolymers, in contrast to harsher detergents, preserve the native lipid environment of MPs, affect their structure to a lesser degree, and allow direct extraction of MPs from biological membranes avoiding the use of detergents at preliminary steps.

Early experiments conducted in the late 1990s–early 2000s suggested a copolymer of styrene and maleic anhydride (SMA) as one of the first amphiphilic agents for direct and efficient solubilization of membrane peptides or proteins [11], which was later extensively optimized and since then became extremely widespread for biophysical and structural characterization of MPs [12,13,14].

The success of SMA has boosted the development of new synthetic copolymers based on SMA and its derivatives. These copolymers are capable of fetching patches of cell membranes into native lipodiscs, the size of which can be fine-tuned. These lipodiscs are stable within a broad range of external conditions (pH, ionic strength, tolerance to divalent ions, etc.).

The progress in this field should ultimately allow researchers to adapt a lipodisc-based technique for isolation and investigation of a vast diversity of MPs and large membrane complexes. Moreover, this approach looks promising in terms of speeding up the high-throughput screening of new compounds in drug discovery as well as determination of the three-dimensional structures of proteins by means of state-of-the-art methods such as cryogenic electron microscopy (cryo-EM and serial femtosecond crystallography (SFX) at X-ray free-electron lasers (XFELs).

In our review, we focus on the present diversity of amphiphilic copolymers capable of membrane solubilization, the molecular mechanisms and efficiency of lipodisc formation, and the benefits of lipodiscs for R&D. The first part overviews the chemical assortment of copolymers, their peculiarities, and effects of both copolymer structure and external factors on the solubilization efficiency and size of lipodiscs. The second part describes the putative mechanisms of lipodisc formation with respect to other types of membrane-solubilizing agents. Finally, in the last part, we discuss the real-world applications and future perspectives of lipodiscs in structural biology.

## 2. Amphiphilic Copolymers Used for Preparation of Lipodiscs

### 2.1. Types of Amphiphilic Copolymers

**Figure 1:**
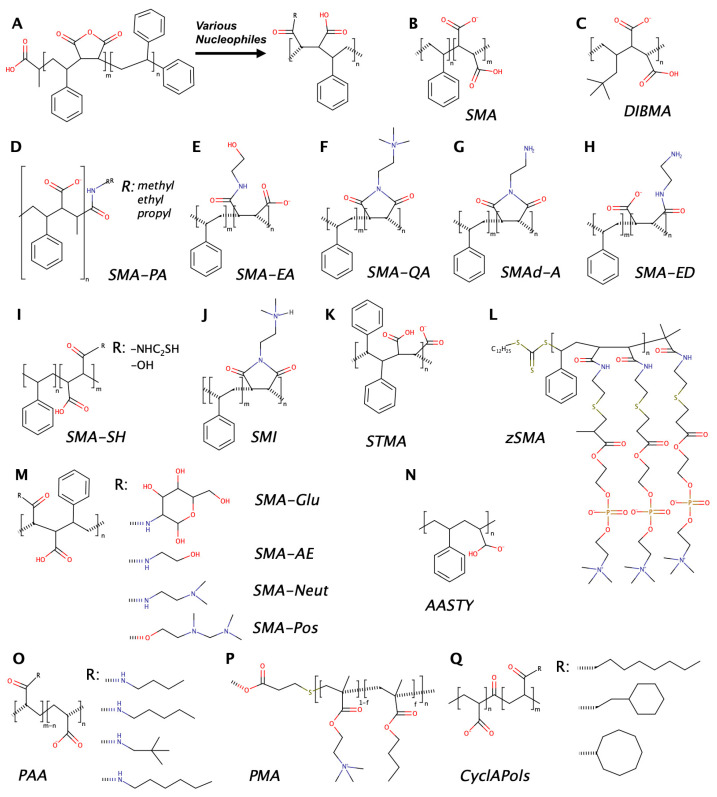
Diversity of amphiphilic polymers capable of forming lipodiscs. (**A**) General scheme for the synthesis of SMAnh derivatives as proposed in [19]. (**B**) SMA polymer; (**C**) DIBMA polymer; (**D**–**Q**) alternative lipodisc-forming amphiphilic polymers. See Table 1 for abbreviations and references.

While styrene–maleic acid (SMA) copolymers are most popular as lipodisc-forming agents, several alternative amphiphilic polymers have been introduced over the recent years. The information about different amphiphilic polymers reported in the literature is summarized in Table 1, and their chemical formulae are provided in Figure 1. Based on the chemical nature, these polymers can be divided into two major groups: derivatives of styrene–maleic anhydride (SMAnh), the precursor of SMA, and diverse non-SMA-based polymers. Below, we will briefly overview some examples belonging to both groups.

Polymers of the first group are the result of modification of SMAnh mainly by means of nucleophilic addition and ring opening (see Figure 1A) [15] at the highly reactive anhydride moiety and are thus termed styrene–maleic anhydride copolymer derivatives (SMADs). Among others, this group includes alkylamine derivatives [16], ethanolamine, ethylene diamine, and styrene maleimide derivatives [17], glucosamine, N,N-dimethylethylenediamine [18], and cysteamine [19]. The rationale behind modifying the original SMA polymer is at least threefold: (1) tuning lipodisc viability at low pH, (2) rendering lipodiscs more tolerant of divalent ions (mainly Ca^2+^ and Mg^2+^), and (3) controlling (at least to some extent) the lipodisc size. Moreover, the cysteamine derivative (SMA-SH) features solvent-exposed sulfhydryl groups readily providing a base for various covalent labeling procedures, including modification with fluorescent probes and biotin, which can be especially useful as it allows avoiding the preparation of engineered membrane protein variants [19].

**Table 1:** Overview of amphiphilic copolymers used for lipodisc preparation. Ð—dispersity calculated as M_w_/M_n_, where M_w_ is the weight average molecular weight and M_n_ is the number average molecular weight.

Polymer Type, Ratio of Hydrophobic:Hydrophilic Units	Mn, kDa	Solubilization Conditions	Ð	Disc Size, nm	Reference
SMA variants					
SMA (Xiran SZ 20010), 4.3:1	2.5	pH = 7–9, Ca^2+^ ≤ 2 mM	2.9	7–10	[20,21]
SMA (Xiran SZ 25010), 3:1	4	pH = 6–9, Ca^2+^ ≤ 2 mM	2.5	7–10	[20,21]
SMA (Xiran SZ 30010), 2.3:1	2.5	pH = 5.5–9, Ca^2+^ ≤ 2 mM	2.6	7–10	[20,21]
SMA (Xiran SZ 40005), 1.2:1	2	pH > 6, Ca^2+^ ≤ 2 mM	2.5	7–10	[20,21]
SMA (RAFT), 2:1	5.4–18	pH > 6, Ca^2+^ ≤ 2 mM	1.28–1.31	27–28	[22]
SMA (RAFT), 3:1	6.4–22	pH > 6, Ca^2+^ ≤ 2 mM	1.25–1.29	9–10	[22]
SMA (RAFT), 4:1	7.4–28	pH > 6, Ca^2+^ ≤ 2 mM	1.25–1.28	31–33	[22]
Styrene−maleic anhydride copolymer derivatives (SMADs)					
SMA-MA, 1:1	5.8	pH > 5, Ca^2+^ ≤ 8 mM	2.5	14	[16]
SMA-EtA, 1:1	6.2	pH = 5–10, Ca^2+^ ≤ 24 mM	2.5	25	[16]
SMA-PA, 1:1	6.5	pH = 5–10, Ca^2+^ ≤ 12.5 mM	2.5	32	[16]
SMA-EA, 1.3:1	2 ^1^	pH > =3.3, up to 21 mM for Ca2+ and 30 mM for Mg2+		10–60	[23]
SMA-QA, 1.3:1	2.1 ^1^	pH = 2.5–10, Ca^2+^ up to 200 mM		10–30	[24]
SMAd-A, 1.3:1	1.8 ^1^	pH < 6, Mg^2+^/Ca^2+^ up to 200 mM		~3–~20	[25]
SMA-ED, 1.3:1	1.8 ^1^	pH > 7 or pH < 5, Mg^2+^/Ca^2+^ 10 (pH = 8.5)- 200 mM		~4–~10	[25]
SMA-SH, 2:1	7.5	stable at pH = 8	polydisp.	11–15	[19]
SMI, 2:1	2.7	pH < 7.8, Ca^2+^ 100+ mM	2.8	6–11	[26]
zSMA, 1:1	12–44	pH > 4, Ca^2+^ up to 20 mM	1.1–1.2	8–30	[27]
SMA-Glu, 2:1	42.1	pH > 3, Mg^2+^ > 100 mM	6.93	10–28	[18]
SMA-Neut, 2:1	6.9	pH = 3–9, Mg^2+^ > 100 mM	1.46	15–60	[18]
SMA-AE, 2:1	18.3	pH = 3–9, Mg^2+^ > 100 mM	1.72	10–28	[18]
SMA-Pos, 2:1	11.1	pH < 3 or pH > 9, Mg^2+^ > 100 mM	1.43	10–28	[18]
SMA-Pos, 3:1	21.9	pH < 3 or pH > 9, Mg^2+^ > 100 mM	1.33	15–60	[18]
Non-SMA-based polymers					
PAA (non-aromatic polyacrylic acid), pentyl-derivative	2.5 ^2^	pH > 6, Ca^2+^/Mg^2+^ < 3.5 mM, 5.5 mM		8–16	[9]
PAA (non-aromatic polyacrylic acid), hexyl-derivative	2.5 ^2^	pH > 6, Ca^2+^/Mg^2+^ < 2 mM, 2 mM		7–14	[9]
PAA (non-aromatic polyacrylic acid), neopentyl-derivative	2.7 ^2^	pH > 6.5, Ca^2+^/Mg^2+^ < 2 mM, 5.5 mM		10–17	[9]
PMA (polymethacrylate)	1.7–14	stable at pH = 5.3–7.3		10–20	[28]
DIBMA, 1:1	8.5–15	pH ≥ 6.5, Ca^2+^/Mg^2+^ ≤ 20 mM	1.4	15–20	[29]
AASTY, 1:~1	6.6–8.9	pH = 6.5+, Ca^2+^ ≤ 7 mM	1.14–1.21	<10	[30]
CyclAPols, 1:1	4.8–5.0	stable at pH = 7	2.0	<40	[31]
STMA, 1:1	4.4–5.8	pH = 5–10, Ca^2+^ ≤ 2.5 mM	1.2–1.5	20	[32]

^1^ derived from 1.6 kDa SMA; ^2^ derived from 1.8 kDa PAA.

While SMADs allow overcoming some limitations of SMA pointed out above, they all are incompatible with certain spectroscopic approaches, e.g., UV absorption and circular dichroism (CD), as they still possess styrene as the hydrophobic unit. Apart from undesirable absorption, styrene can be also involved in π–π or π–cation interactions with the embedded protein or lipids [28]. Several non-SMA-based alternatives have been proposed to solve these problems. Probably the most extensively explored one is the diisobutylene–maleic acid (DIBMA) copolymer [29], in which the aromatic styrene unit is replaced with aliphatic diisobutylene. A few other polymers of this type have been recently developed [9,28,31].

Finally, a number of amphiphilic polymers still containing a styrene block but not directly related to SMA have been also proposed, demonstrating higher protein extraction efficiency as in the case of AASTY [30] or better homogeneity and larger lipodisc size as in the case of STMA [32].

### 2.2. Influence of Polymer Concentration, Type and External Factors on Lipodisc Size and Solubilization Efficiency

The type of amphiphilic polymer as well as its specific formulation and solubilization protocol can greatly influence solubilization efficiency and the size of resulting lipodiscs. Below we will discuss some factors that can affect the ability of different polymers to form lipodiscs and lipodisc size. However, it is important to preface the further discussion with the note that the size of lipodiscs may vary depending on the method used for the measurements [3]. The size of lipodiscs obtained using TEM may appear larger than their actual size due to negative staining. Similarly, dynamic light scattering (DLS) and size-exclusion chromatography (SEC) tend to overestimate the size because of the hydration shell present around lipodiscs. Small-angle X-ray scattering (SAXS) seems insensitive to these limitations, estimating size more accurately [26].

#### 2.2.1. Effect of Polymer Concentration

One of the prominent factors affecting lipodisc size is the lipid:polymer ratio. As a general rule of thumb, a higher proportion of polymers with respect to lipids results in smaller lipodiscs. A possible explanation for this behavior may be the tendency to maximize the total perimeter of lipodiscs due to the formation of favorable interactions between polymers and lipids. Thus, higher concentrations of polymers yield smaller lipodiscs with a longer total perimeter.

This rule is fulfilled for DIBMA, which forms homogeneously sized lipodiscs with a diameter of ca. 20 nm at a polymer:DLPC molar ratio of 1:10 and of ca. 12 nm (i.e., almost two times smaller) at a ratio of 1:4 [29]. Another systematic study of the dependency of DIBMALP size on polymer concentration was recently reported in [33], indicating that DIBMALPs with a diameter of up to 40 nm can be obtained at the 1:12 polymer:lipid molar ratio corresponding to the lowest examined DIBMA concentration. It is noteworthy that the size homogeneity of DIBMALPs remained unchanged regardless of the polymer:lipid molar ratio.

Similar concentration dependence was observed for alkyl-PPA polymers: the increase in the polymer:lipid weight ratio by a factor of 5–7 (from 1:5 to 1.5:1) results in the decrease in the lipodisc size by a factor of 2 (from ~14–17 nm to ~7–10 nm depending on the hydrophobic group) [9].

For SMA-QA, SMA-ED, and SMAd-A, the trend is similar [25]. SMA-QA forms 30 nm lipodiscs taken in 1:4 polymer:lipid ratio while the 1:1.5 ratio results in 10 nm lipodiscs [24]. The same effect was also observed for PMA [28] and, to a somewhat lesser degree, SMI [26] polymers.

Popular protocols for SMA-mediated solubilization consider intentionally high polymer:lipid ratios, e.g., ~1:3.7 SMA:lipid molar ratio (i.e., 3:1 *w*/*w* ratio) [20,34] and even higher 1:1.25 molar ratio [22]. By analogy with DIBMA, such a polymer:lipid ratio should yield lipodiscs with a diameter that is at the lower boundary of the size range. Indeed, Zhang et al. assessed the dependence of lipodisc size on the polymer concentration in [35], showing that lower polymer:lipid ratios, e.g., 0.75:1–1.75:1 *w*/*w*, resulted in the formation of larger lipodiscs with a diameter of up to 31 nm.

#### 2.2.2. Effect of Polymer Length and Molecular Weight

The possibility to modulate the lipodisc size by the molecular weight of amphiphilic polymers was proposed in [17] by analogy with membrane scaffold proteins (MSPs). The high-molecular-weight MSPs do not allow for size control, forming nanodiscs with fixed dimensions, while lower-molecular-weight peptides have been demonstrated to enable size control [36,37]. In agreement with this hypothesis, the size of lipodiscs prepared using SMA-EA with low molecular weight (1.6 kDa) could be tuned in the range from 10 to 60 nm by varying the polymer:lipid ratio [17].

While a number of SMA fractions with different molecular weights spanning the 1.1–6.5 kDa range were obtained and scrutinized in [38], no similar trends for the lipodisc size were observed. SMA polymers of all tested molecular weights produced lipodiscs with a diameter of ~7–10 nm. At the same time, low-molecular-weight polymers were found to be the most efficient in membrane insertion and membrane solubilization, and the lipids in such lipodiscs were found to exhibit faster exchange rates between lipids in neighboring lipodiscs, suggesting that low-molecular-weight SMA polymer causes more disruption of native lipid–lipid interactions, thus decreasing thermodynamic stability. No variations of lipodisc size were observed for SMA polymers of low (5.4–7.4 kDa), medium (8.5–16 kDa), and high (18–28 kDa) molecular weight in [22]. Importantly, in both studies, a single polymer:lipid ratio was tested. As noted in the previous section, the lack of data about how the SMA:lipid ratio may influence the resulting lipodisc size leaves room for speculation whether low-molecular-weight SMA taken at different concentrations can yield larger lipodiscs.

The size of lipodiscs prepared using the RAFT-made DIBMA with molar weight varying from 1.2 to 12 kDa appears confusing [39]. While TEM results did not reveal any clear size dependence on the molecular weight, the complementary DLS analysis uncovered that DIBMA polymers with higher molecular weight result in smaller lipodiscs. It should be noted that DLS provides an integral evaluation for the size of all particles present in a solution. At the same time, the solubilized samples consist of a mixture of lipodiscs, polymer aggregates, and disrupted vesicles. Thus, the inhomogeneity of samples may possibly explain the observed discrepancy between TEM and DLS results and the bias of the latter towards larger particle sizes [22]. Again, only a single polymer:lipid ratio was considered in this study, precluding further evaluation of the concentration dependency.

For zwitterionic zSMA polymers, the lipodisc diameter can be modulated simply by using polymers of different molecular weight at the same polymer:lipid ratio. While the 12 kDa polymer formed lipodiscs with an average diameter of 8 nm, the 44 kDa polymer allowed for 30 nm lipodiscs [27].

While some low-molecular-weight polymers are able to efficiently form lipodiscs and even allow the control of their size in a concentration-dependent manner, a couple of studies imply that at least for some polymers there exists a lower molecular weight threshold for membrane solubilization. For DIBMA, it equals 1.2–1.3 kDa [39]; for PMA (polymethacrylate), ~3 kDa [28]. Computer simulations of short SMA (~1.3 kDa) also imply its inability to solubilize membranes [40]. In terms of the contour length of polymers, these molecular weight values correspond to ca. 5–8 nm. On the other hand, a number of studies suggest that solubilization by very large polymers may also be less effective due to steric hindrance in longer polymers and their tendency to form aggregates [41]. Particularly, an upper molecular weight limit of 10 kDa was earlier proposed for optimal solubilization [42,43].

#### 2.2.3. Effect of Mono- and Divalent Ions

The increase in ionic strength of the copolymer solution by addition of monovalent ions (most commonly NaCl) results in electrostatic screening, which reduces repulsion both between membrane-adsorbed/free charged copolymers and lipodiscs and thus may influence the formation and the dynamics of lipodiscs. Particularly, increasing the NaCl concentration accelerates the formation of SMALPs [20,34], increases the yield of protein extraction by SMA [44], and speeds up collisional lipid transfer among SMALPs [45]. Similar effects of monovalent ions were observed for DIBMA-stabilized lipodiscs [46,47].

Divalent ions, predominantly Ca^2+^ and Mg^2+^, are essential for a number of enzymes and other proteins, including ion channels [48,49,50] and GPCRs [51]. At the same time, these ions tend to be chelated by carboxylate groups and at some concentration lead to precipitation of carboxyl-containing polymers/lipodiscs, apparently because Ca^2+^ and Mg^2+^ ions induce conformational strain or a conformational change in the polymer [42,52,53]. The most notable effect is observed for SMA, which does not tolerate the concentration of Ca^2+^ over 1 [42] or 2 mM [20,21], though a bit higher tolerable concentration of Mg^2+^ (up to 4 mM) has been shown for SMA 2000 (Cray Valley) in [42]. Interestingly, the divalent ion tolerance may depend on the lipodisc size as larger lipodiscs formed by SMA 2000 are stable up to 5 mM MgCl_2_ while smaller ones formed by SMA 3000, XZ09008, and SZ25010 precipitate at a lower concentration, ≤1 mM [42]. It should be noted however that the observed dependence may rather be related to different polymer formulations/types than to the lipodisc size itself.

SMA derivatives with a single carboxyl group remaining in each maleic acid unit demonstrate higher tolerance to divalent ions: up to 8 mM for SMA-MA, 12.5 mM for SMA-PA, 24 mM for SMA-EtA, and 20–30 mM for SMA-EA. Comparing the family comprising the first three of these polymers (SMA-MA, SMA-PA, and SMA-EtA), Esmaili et al. hypothesized that longer and more flexible propyl groups of SMA-PA can branch out and thus become more accessible to hydrophobic interactions as compared with shorter ethyl groups of SMA-EtA, resulting in the observed nonlinear dependence of the tolerable bivalent ion concentration on the sidechain size [16].

The modification of maleic groups (for example, esterification) certainly leads to increased tolerance to divalent ions in many cases, as pointed out in the previous section. However, there exists at least one counterexample. SMA with maleic acid residues partially esterified with 2-butoxyethanol demonstrated increased sensitivity to Mg^2+^ [53,54] as compared to unmodified SMA. A possible explanation for this behavior might be similar to the one proposed for SMA-PA as the resulting pending moiety is rather long, flexible, and hydrophobic (see the previous paragraph).

Interestingly, for SMA-EA, the highest attainable Ca^2+^/Mg^2+^ concentration increases with the increase in polymer concentration corresponding to the decrease in lipodisc size, from 9.3/10 mM at 1:1 DMPC:SMA-EA *w*/*w* ratio up to 21.3/30 mM at 1:3 ratio [23]. This is in contradiction with the inverse dependence of Mg^2+^ resistance of SMALPs on their size reported in [42], which, however, must be taken with caution for the reason stated above.

Zwitterionic SMA-ED does not precipitate at the Ca^2+^ concentrations up to 10–200 mM depending on pH [25], while another zwitterionic polymer, zSMA, is stable at least up to 20 mM of CaCl_2_ [27]. At the same time, the positively charged SMA derivatives, SMAd-A [25] and SMA-QA [24], lacking carboxyl groups entirely, remain functional at least up to 200 mM.

DIBMA, which contains maleic acid blocks similar to SMA, can tolerate a somewhat higher concentration of both Ca^2+^ and Mg^2+^ up to 20 and 25 mM [29], which may be attributed to the shift of carboxyl pKa values and thus to a higher ratio of protonated carboxylates due to a higher local density of carboxylates in this polymer. At the same time and in contrast to SMA, the addition of divalent ions accelerates DIBMA-mediated solubilization of membranes, increases its efficiency, and speeds up lipid transfer among DIBMALPs while preserving their morphology. These effects appear more pronounced compared to the similar effects of monovalent ions discussed above and thus cannot be simply attributed to the electrostatic screening, but they are rather related to the specific interactions between divalent counterions and DIBMA [55].

Apart from the above-mentioned effects of ions on the solubilization efficiency and kinetics, the increase in concentration of both mono- and divalent ions considerably decreases the size of lipodiscs formed by DIBMA. Particularly, the presence of 10 mM Mg^2+^ and Ca^2+^ reduced the diameter of DIBMALPs to on average 32 (±18) and 23 (±15) nm, respectively, as compared to 46 (±26) nm in the absence of these ions [55]. The same effect was achieved by adding 200 mM NaCl [46]. Again, the more pronounced effect for Ca^2+^ as compared to Mg^2+^ implies that it is not solely due to enhanced electrostatic screening. We did not find similar data on the effect of the ionic strength on the size of SMALPs; however, it is known that higher ionic strength decreases the solubility of SMA and thus can ultimately affect the solubilization efficiency of this polymer as well [20].

#### 2.2.4. Effect of pH and Polymer Charge

The hydrophilic units of many amphiphilic copolymers bear acidic carboxyl/carboxylate groups. Alteration of the pH of a solution results in their reversible protonation and changes the polymer’s total charge, which in turn affects its structure and solubility. While the pKa values of the two carboxyl groups in a maleic acid unit of SMA are equal to ~6 and ~10 [56], the actual values for different variants of SMA may vary in the ranges of 4.4–5.9 for the first group and 8.6–9.0 for the second [20]. Therefore, pH below the lower pKa leads to almost complete protonation of SMA, fading of electrostatic repulsion between the carboxylate groups, and, consequently, its excessive aggregation and precipitation [3].

The inability of SMA to solubilize membranes at pH below 5.5 could affect several biochemical assays and limits the applicability of this polymer to the proteins requiring low pH to attain their physiological state. This drove the development of alternative polymers either with zwitterionic/positively charged units (e.g., SMA-Neut, SMA-Pos [18]) or lacking the acidic groups entirely (e.g., zSMA [27]), broadening the pH range suitable for membrane solubilization.

It is worth mentioning that the pH sensitivity may be more complex, as in the case of SMA-ED, which is stable under all pH values except pH = 5–7. This is apparently due to the zwitterionic nature of this polymer in this pH range leading to its hypercoiling and aggregation due to intramolecular charge–charge interactions [25].

A quite distinct problem related to the polymer charge is its interplay with the charge of soluble domains of lipodisc-reconstituted proteins. This question was specifically addressed in [57]. The authors evaluated the solubilization efficiency of both positively and negatively charged proteins (cytochromes P450 and b5, respectively) by means of negatively charged SMA-EA and SMA and positively charged SMA-QA. They revealed that SMA-EA- and SMA-based lipodiscs inactivated cytochrome P450 during reconstitution due to charge–charge interaction between the negatively charged polymer and cationic protein. The authors concluded that the compatibility between polymer and highly charged proteins should be carefully considered when planning structural studies.

#### 2.2.5. Effects of Monomer Size and Chemical Nature

Overall, a careful meta-analysis of how the properties of monomers affect solubilization efficiency and lipodisc parameters is restricted since it is usually difficult to separate the effects imposed by the monomer nature from other factors, including polymer size and concentration as well as external factors, when comparing different studies. A valuable exception is a recent attempt to assess the solubilization efficiency of a large library of amphiphilic polymers reported in [41]. The authors highlight that the optimal balance of such parameters as amphiphilicity, flexibility, and size of pendant groups may be very delicate, and both upper and lower extremities in the values of these parameters are not optimal for the best performance. For instance, the polymers (primarily, their backbone) should be sufficiently flexible to expose their hydrophilic groups to the aqueous phase and their hydrophobic groups to the lipid acyl chains. At the same time, extreme flexibility (especially of the pendant groups) is not beneficial as it leads to higher entropy loss upon the membrane insertion, as supported by a less effective solubilization of membranes induced by poly(ethyl)acrylic acid (PEAA) as compared to SMA [34,58,59]. A similar balance is required for the size of pendant groups. Smaller groups allow for better membrane insertion. However, polymers with such groups may be less efficient in later steps of solubilization (see the appropriate section below), i.e., disruption of the lipid packing, where bulkier groups perform better.

The survey of both polymers with charged polar groups (SMA-Glu, SMA-AE) and uncharged or zwitterionic ones (SMA-Neut) suggested that the latter resemble softer surfactants resulting in milder effects on solubilized proteins and larger lipodiscs [18]. The authors also hypothesized that the presence of longer styrene tails at the end of SMA as in the case of SMA 3:1 endows this polymer with softer surfactant properties resulting in larger lipodiscs. Alternatively, the increased lipodisc size might be attributed to the longer persistence length of SMA 3:1 polymers as longer styrene blocks may stiffen polymers due to steric clashes between bulky styrene groups. A similar connection between the persistence length of STMA copolymers (which is 30–50% greater than those of SMA analogs, i.e., ~2.2 nm) and twice bigger lipodiscs as compared to SMALPs according to TEM has been proposed in [32]. It should be noted, however, that while the persistence length and the intrinsic curvature of polymers can control the lipodisc size to some degree, this influence is relatively flexible, allowing the incorporation of membrane proteins through the formation of larger lipodiscs that is seen in the lipid-only systems (discussed in the next section) [60].

At the same time, bulkier and branched hydrophobic groups increase the disorder of lipids located close to the polymer belt of the lipodiscs, as indicated by shifted phase transition temperatures of lipids in lipodiscs stabilized by hexyl- and branched neopentyl-PAA compared to pentyl-PAA [9], indicative of a strong bilayer perturbation.

#### 2.2.6. Effects of Embedded Proteins

Apart from polymer properties and external factors, the nature of solubilized proteins may also influence the size of lipodiscs. It was reported that SMA yielded larger lipodiscs when a membrane protein was embedded [34]. Incorporation of a photosynthetic reaction center [61], the mitochondrial respiratory complex IV [62], and bacteriorhodopsin [63] resulted in lipodiscs with a diameter between 12 and 17 nm, while solubilization of the staphylococcal penicillin-binding protein complex PBP2/PBP2a even led to lipodiscs up to 24 nm in diameter [64]. Apparently, the protein content, and most likely specifically its shape and diameter, are key parameters that determine the size of resulting nanodiscs.

#### 2.2.7. Effects of Lipid Types and Phase

The size of lipodiscs formed by SMA does not significantly change upon variation of length of the lipid acyl chains; however, the solubilization kinetics depends on their saturation [34]. Unsaturated lipids are more difficult to solubilize than saturated lipids in the fluid phase. The authors explained this quite counterintuitive fact by increased lateral pressure in the acyl chain region of membranes composed of unsaturated lipids resulting in a less efficient insertion of SMA.

The same study reports that solubilization was improved by elevated temperature as a rule of thumb [34] with a single exception for membranes containing anionic saturated lipids that have long acyl chains in the liquid-crystalline phase and at low ionic strength. Most likely, this is a direct consequence of the low membrane binding of SMA, due to strong unscreened repulsive electrostatic interactions in this particular case.

## 3. Formation, Structure and Dynamics of Lipodiscs

Direct structural details on how SMA and related copolymers solubilize lipid vesicles in solution remain elusive. However, a combination of experimental and computational approaches has shed some light on this process recently. It is generally believed that it consists of three consequential although tightly intertwined steps: (1) polymer binding to membrane, (2) insertion of polymers into the hydrophobic core of a membrane, and (3) eventual solubilization of membrane and formation of lipodiscs [3,20,34]. We will discuss these steps with the focus on the structural details largely available for SMA, which is the most extensively studied type of lipodisc-forming polymer. Moreover, we will also compare putative mechanisms of bilayer solubilization by amphiphilic copolymers with other membrane-solubilizing agents.

### 3.1. Membrane Binding

Amphiphilic polymers form aggregates in aqueous solution. These aggregates correspond to globular, collapsed copolymer conformation identified for SMA by SAXS experiments [65] and coarse-grained simulations [40]. Nile red fluorescence experiments indicated that this conformation containing hydrophobic domains allows for the most effective solubilization as opposed to the random coil conformation [20]. A number of factors may influence the morphology and the extensiveness of polymer aggregates such as the charge and concentration of polymers, the pH and ionic strength of the solution, and the degree of polymer blockiness [40,65]. In turn, all of these factors may eventually affect the effectiveness of solubilization [3]. Generally, less charged polymers with higher ratio of hydrophobic monomers (styrenes for SMA) form more compact clusters [40]. It is also important to note that while the formation of relatively compact clusters of polymers is favorable for the consequent membrane solubilization, the excessive aggregation of polymers, e.g., at pH values close to those rendering them too hydrophobic due to protonation of the carboxyl groups, prevents efficient solubilization. Particularly, it may occur because of the competition between polymer–polymer and polymer–lipid interactions in the situation of high local density of polymers at the membrane surface. These polymer–polymer interactions may even induce aggregation of the polymer-coated lipid vesicles [20].

Interaction between polymers and lipid membranes itself proceeds from the complex interplay between hydrophobic binding, Coulombic contributions, self-assemblies of polymers and lipids, and possible additional effects (e.g., effect of structural constraints) [66].

Overall, it is believed that the hydrophobic interactions between the hydrophobic polymer units and the acyl chains of lipids [67,68,69] are the main driving force at the initial step of membrane solubilization, which is strong enough to overcome the electrostatic repulsion between negatively charged SMA copolymer and bilayers consisting of anionic lipids even at increased surface pressures [34]. Computer simulations of SMA also indicate that polymers readily bind to the membrane and penetrate into its hydrophobic core starting with the hydrophobic (styrene) moieties of the SMA polymeric termini [8]. It is also worthwhile noting that for a number of various polymers carrying several hydrophobic anchors, the binding appears significantly more stable [70,71,72,73,74,75].

Still, electrostatic interactions can modulate binding. Increased ionic strength, leading to better charge screening, and a lower fraction of anionic lipids in the bilayer both lead to higher efficiency of solubilization by apparently improving the polymer binding [34]. Moreover, a certain favorable contribution of electrostatic interactions cannot be excluded. Since the lipid bilayer possesses positive potential in its hydrophobic core while the pendant phenyl groups of SMA have a large quadrupole moment with a negative potential on both sides of the aromatic ring [76,77], it was suggested that they can favor the SMA insertion inside the membrane [34] and/or its deeper insertion, as reviewed in the next section.

### 3.2. Insertion of Polymers into a Membrane and Membrane Solubilization

Following the initial fast adsorption at the membrane surface occurring at the timescale of 10–500 ns according to computer simulations [8,40], amphiphilic polymers penetrate deeper into the hydrophobic core, further destabilizing the membrane. The presence of packing defects in the membrane is thought to facilitate this process and to enhance solubilization eventually. It is strongly supported by observations that the lipid phase affects the solubilization efficiency. For the membranes consisting of saturated lipids, the solubilization efficiency reaches its maximal extent at the phase transition (Tm) temperature of lipids, where the number of packing defects increases due to the coexistence of the liquid gel and the liquid crystalline phases [34]. On the other hand, the membranes consisting of lipids with longer acyl chains tend to be more resistant against solubilization by SMA simply due to tighter contacts between lipids. Although unsaturated lipids render the bilayer less ordered in general, the solubilization efficiency is reduced in this case presumably because of increased lateral pressure in the acyl chain region of membranes with such lipids which hampers the insertion of polymers [34].

Several scenarios have been suggested for the next step of the membrane disintegration process upon the insertion of polymers. According to the first hypothesis, originally proposed in the theoretical study of 2:1 SMA copolymers [8], the amphiphilic polymers accumulate at the membrane surface and eventually induce the formation of hydrophilic water-filled pores with polymers lining the pore edge. Subsequently, the pores gradually increase in size up to the diameter of 5–10 nm. However, the complete destruction of bilayer and formation of lipodiscs was not observed in that study, presumably due to the limitations of the simulation setup (application of the periodic boundary conditions). Another recent computational study supported the ability of SMA to stabilize transmembrane pores [78]. The pore-forming activity of SMA was also experimentally confirmed in [65] and [79]. The authors of the latter study have further speculated, suggesting that the SMA-induced membrane pores eventually merge to form a lipid island, which then moves out of the bilayer as a mature lipodisc.

In another study, the interactions of both 2:1 and 3:1 SMA copolymers of different monomer sequences with lipid bilayers were modeled [40]. It was found that while 2:1 SMA interacted with membranes in the same way as in [8] resulting in porous membranes, the aggregates of 3:1 SMA existing in solution were capable of pulling patches of lipids out of the bilayer plane. It was assumed that such behavior depends on the presence of hydrophobic units with ≥3 sequential styrene monomers, which act as drivers for the formation of lipodiscs. Presumably, such extended styrene units maintain the integrity of SMA aggregates and, at the same moment, are able to efficiently replace hydrophobic interactions between the acyl chains of lipids by their interactions with the hydrophobic core of SMA aggregates.

**Figure 2:**
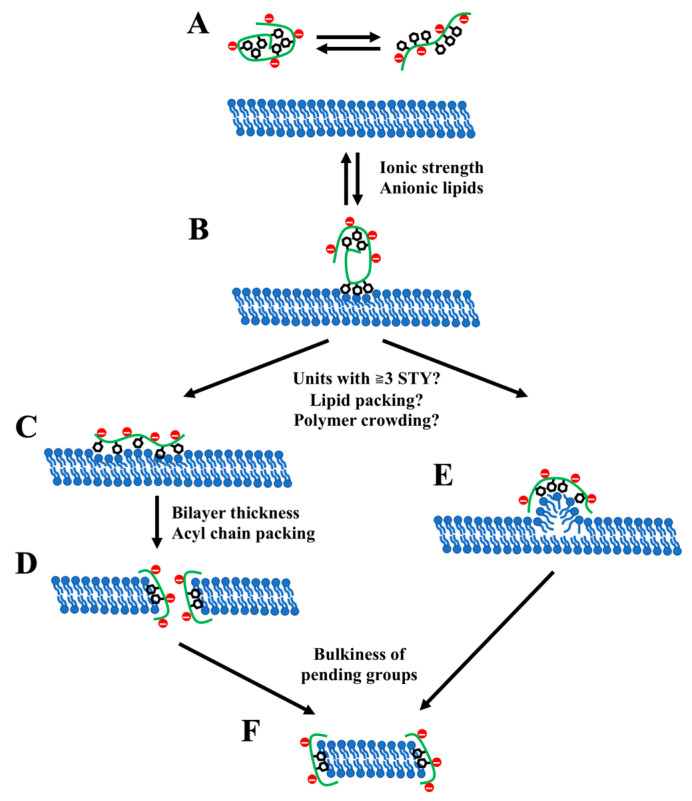
Putative mechanisms of SMA-mediated solubilization of membranes. Polymer is colored green, styrene groups are schematically shown as black hexagons, and maleic acid residues are shown as red circles. (**A**) Equilibrium of extended SMA and compact SMA aggregates in solution; (**B**) initial absorption of SMA on the bilayer; (**C**,**D**) membrane solubilization via the SMA-induced poration (“pore” mechanism); (**E**) membrane solubilization via the pulling of lipid patches by SMA aggregates (“extraction” mechanism); (**F**) formation of mature lipodiscs.

Despite the obvious difference between the two proposed mechanisms (see Figure 2 for the schematic overview), they can coexist, prevailing one over the other depending on, e.g., the specific conditions or the type of polymer. As pointed out above, the fraction of prolonged styrene repeats can be among such factors [40]. Polymers with a high fraction of such units are able to form lipodiscs by the second (“extraction”) mechanism, while when the fraction of extended hydrophobic units is low, the SMA solubilizes membranes mainly by the first (“pore”) mechanism.

One can see an analogy with an apparent lack of a single mechanism of membrane solubilization, as previously suggested for classical detergents [80]. Depending on the detergent transmembrane swapping time (i.e., the flip-flop time), which affects its partition between lipid layers, two possible modifications were proposed [81,82,83] to the classical three-state model of Helenius and Simons [84]. In the case of the fast flip-flop, the detergent rapidly distributes between lipid layers, leading to a solubilization process via open vesicular intermediates [83]. Contrarily, in the slow flip-flop limit, the detergent cannot efficiently move across the membrane, and the system goes through a nonequilibrium state caused by detergent mass imbalance in the outer leaflet [80]. In this case, the solubilization occurs via the binding of detergent micelles to the bilayer with an extraction of lipids and/or formation of mixed lipid/detergent aggregates [81,85]. The prevalent way of solubilization depends, primarily, on the lipid packing and detergent type [82,83]. Moreover, both mechanisms can co-occur. Partial solubilization starts from the slow extraction of lipids from the outer leaflet causing detergent leakage inside the liposome followed by transmembrane equilibration of detergent and subsequent micellization through the fast bilayer-saturation mechanism [85].

While amphiphilic copolymers are generally considered as milder solubilizing agents [31] as compared to low-molecular-weight detergents, certain parallels between their mode of action and those of classical detergents inevitably arise. Indeed, the fast-saturation mechanism described for detergents may share similarity with the “pore” mechanism of membrane solubilization suggested for SMA, while the “extraction” mechanism of SMA-mediated solubilization resembles the slow flip-flop limit of detergent-mediated solubilization. It is worth emphasizing that the above example of classical detergents further promotes the hypothesis that either two alternative mechanisms can co-occur or one of them can predominate over the other depending on the membrane and polymer composition or external factors affecting, e.g., the lipid packing.

Apart from the above-mentioned parallels between the classical detergents and amphiphilic copolymers, the two putative mechanisms of SMA-mediated membrane solubilization also resemble the alternative mechanisms of action commonly considered for antimicrobial peptides, i.e., pore and carpet models [86], which were also used to explain the interactions between artificial polymers and biological membranes [87,88]. Particularly, the carpet model (which implies that clusters of amphiphilic peptides crowd on the membrane surface, resulting in its destabilization and eventual solubilization [89]) may resemble to some extent the “extraction” model proposed for SMA in [40]. Overall, it suggests that various amphiphilic agents capable of membrane remodeling may share striking similarity in their modes of action. Future works on scrutinizing the molecular mechanisms of membrane solubilization by lipodisc-forming copolymers as well as the development of novel types of such copolymers can benefit from considering these parallels.

### 3.3. Formation of Lipodiscs and Lipodisc Morphology

At the final step, the destabilized membrane further falls apart with the formation of mature lipodiscs. It is believed that the bilayer thickness plays an important role at this stage, as confirmed by observations that thicker membranes with longer acyl chains are more difficult to solubilize. This is likely due to an increased strength of hydrophobic interactions maintaining the integrity of the bilayer, which must be overcome in order to break up the membrane [3,34]. In line with these observations, the short-chained saturated lipids are easily solubilized, even in the gel phase, while the unsaturated lipids at lower temperature are less efficiently solubilized due to the increased effective length of the lipid acyl chains [34]. On the other hand, copolymers with bulkier hydrophobic pending groups may be more efficient in disrupting lipid packing and thus promoting membrane solubilization [41].

**Figure 3:**
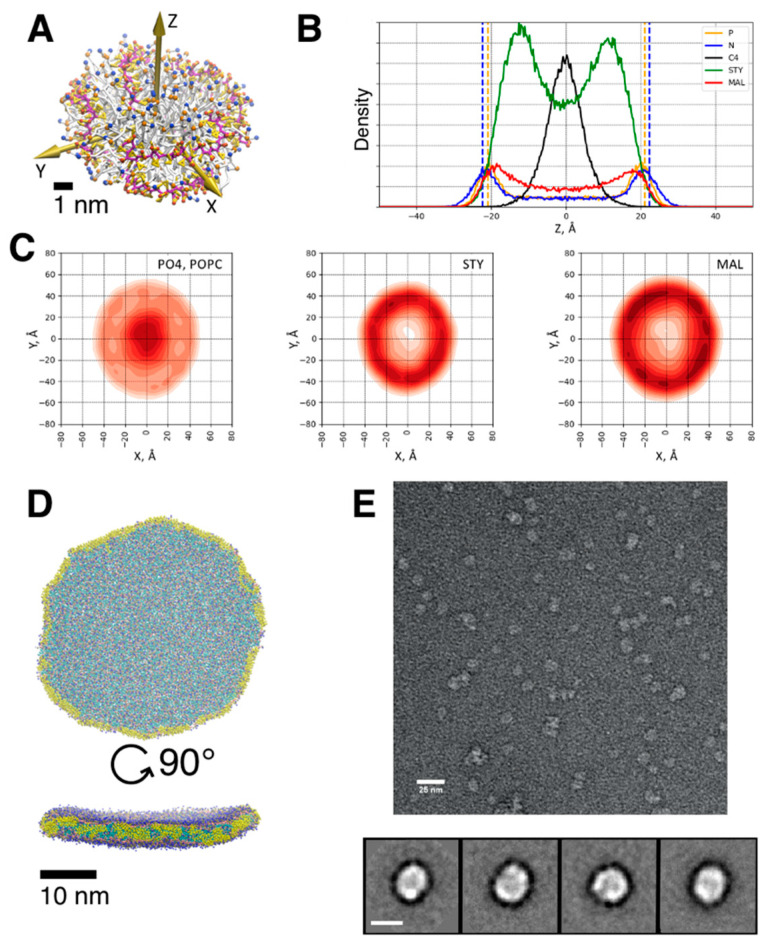
Molecular morphology of lipodiscs. (**A**) Coarse-grained model of SMALP with its principal axes of inertia aligned with the coordinate axes. The model was adapted from [69]. Phosphate, choline, and maleic acid (MA) moieties are shown as orange, blue, and red spheres, respectively; styrene rings in SMA are shown as yellow triangles/spheres; and backbones of SMA copolymers and lipids are shown as gray and white sticks, respectively. (**B**) Number density profiles along Z-axis for POPC phosphate (P), POPC choline (N), terminal groups of POPC acyl chains (C4), styrene (STY), and maleic acid (MAL) residues of the SMALP shown in panel A. Dashed lines indicate the average position of phosphates and choline moieties in the pure POPC bilayer. (**C**) Density maps in XY plane plotted for phosphates (left), styrene (center), and malate (right) residues of the SMALP shown in panel A. (**D**) Coarse-grained model of DIBMALP. DIBMA, choline, phosphate, and acyl chain moieties are shown as yellow, blue, green, and cyan spheres [93]. (**E**) TEM image of the empty SMA lipodiscs containing POPC lipids along with the representative examples of two-dimensional class images. The scale bar for the classes corresponds to 10 nm.

The overall disc-shaped morphology of mature lipodiscs regardless of the type of copolymer used for their preparation has been confirmed by a number of experimental techniques, including electron microscopy, atomic force microscopy [90,91], and small-angle scattering [67,92] techniques, and computer simulations [8,93,94] (see Figure 3). Both experiments and computer simulations indicate that the lipid patch is wrapped in a single layer [3] of polymer similar to nucleic acids in the histone complex or membrane-scaffolding protein in nanodiscs. Polymers form two distinct belts in the case of SMA and a single broader belt in the case of DIBMA [69]. The styrene moieties of SMA are placed between the lipid acyl chains of lipids while the hydrophilic maleic acid residues face the solution according to FTIR and NMR experiments [22,67,95]. Recent SAXS data, however, indicate that apart from the polymer belt at the rim of lipodiscs, some SMA may also remain adsorbed on the surface of lipodiscs [65]. The thickness of lipodiscs ranges from 4.5 to 5.8 nm depending on the bilayer content and agrees well with the expected thickness of intact bilayer in the fluid phase [3].

### 3.4. Dynamics of Lipids in Lipodiscs

The internal dynamics of lipids (their lateral and rotational diffusion, the order of lipid tails, etc.) in lipodiscs could be affected by interactions between the lipid molecules and the encircling copolymer, which justifies investigation of the dynamic properties of the copolymer-enclosed lipid bilayers.

Killian and coworkers studied to what extent the SMA belt can affect the order and dynamics of the enclosed lipids using azobenzene-labeled phospholipids [96]. The study demonstrated that isomerization of azolipids incorporated in SMA-stabilized nanodiscs upon exposure to light of 365 nm is not hindered, indicating that SMA polymers behave as rather flexible belts and allow expansion of the enclosed lipid material. The observed dynamic character also corresponds to the data on collisional lipid exchange [38,97] and on the polymer exchange [98] between lipodiscs.

Detailed information on the lipid dynamics could be obtained by electron paramagnetic resonance (EPR) spectroscopy of specifically placed spin labels [99,100] enabling the investigation of interactions between lipids and MACPs in lipodiscs [69,101,102,103,104]. Specifically, the EPR spectral line of the PCs spin-labeled at different depths of the lipid bilayer was broader in DMPC and POPC particles encased by SMA polymers as compared to that in DMPC and POPC vesicles. It was suggested that lipid dynamics could be constrained by SMA polymers [95,104]. The EPR spectroscopy data on DMPC particles showed that the SMA (3:1) copolymer exerts lateral pressure on the lipid tails. Therefore, carbon atoms at certain positions in the acyl chains were restricted in their motion. These findings indicate that the SMA copolymer stabilizes the lipid bilayer patch by encasing its hydrophobic core [95]. EPR measurements also detected the effect of RAFT-synthesized SMALPs on lipid chain mobility. The EPR spectra showed higher rigidity at the 12th position as compared to the spin labels attached at the 5th or 16th position [104].

A comparison of the lipid dynamics in lipodiscs formed by SMA or DIBMA copolymers showed that the lipids were dynamically more constrained in SMALPs than in DIBMALPs [69]. Complimentary CG MD simulations revealed that DIBMA copolymers form only one lipodisc-encircling belt unlike two such belts in SMALPs. The lipid dynamics in the presence of only one DIBMA belt resembled those in liposomes. Further investigations demonstrated that the dynamics of DIBMALP-enclosed lipids were not altered by the size of DIBMALPs [33]. The lipid dynamics only slightly increased in the presence of the membrane protein sensory rhodopsin II of *Natronomonas pharaonis* (*Np*SRII) as compared to empty DIBMALPs [93].

EPR spectroscopy of spin-label doxyl moieties incorporated into the lipid bilayer in the 5th or 16th position combined with differential scanning calorimetry (DSC) allowed characterizing the temperature-dependent lipid properties in a DMPC model membrane surrounded by SMA, DIBMA, or poly(styrene-co-maleic amide sulfobetaine) (SMA-SB), as compared to liposomes [105]. The authors found that all three polymers broadened the main melting transition of DMPC, changed the water accessibility within the lipid bilayer, and imposed additional constraints onto the lipids. In the case of both SMA and SMA-SB, the rotational mobility of spin-labeled lipids decreased, whereas DIBMA exerted fewer restraints, probably due to its aliphatic side chains. Furthermore, effects of both SMA and SMA-SB could be observed for lipids within myelin-like nanodiscs in the hydrophobic center of the bilayer and near the carbonyl groups. The copolymers exerted steric constraints onto the hydrophobic part of the lipids, while a small loosening effect was observable for the carbonyl-near membrane region [106]. The authors concluded that the choice of the solubilizing polymer is important in forming lipodiscs [105].

## 4. Applications of Lipodiscs in Structural Biology

SMA and related copolymers found many diverse applications in structural biology. First of all, they were efficient in solubilization of a wide range of membrane proteins, including GPCRs [107,108,109,110,111,112], ABC transporters [113,114,115,116], ion channels [117,118,119], photoreaction centers [61,120,121], and electron transport chain complexes [62], expressed in bacteria [42,122,123,124,125], yeast [126,127,128,129], insect [116,130,131], and mammalian cells [115,132,133], as well as plants [134]. As reviewed by Overduin and Esmaili, SMA is effective for solubilizing both monomeric and oligomeric proteins as well as those that are unstable, low-abundance, or lipid-dependent [13]. Extraction using polymers can be used to study specific interactions of membrane proteins with other membrane proteins [13], with lipids [122,135,136,137,138], and with ligands or substrates [115,116,126,128,139] and even to study phage–host interactions [140]. Following extraction with SMA, MPs can later be reconstituted back into lipid bilayers for functional studies [119,122]. Proteins embedded in SMALPs can be further studied using a range of techniques, including mass spectrometry [135,137,138,141] and spectroscopy [115,116,122,126,128,139,142], and various structural methods [143], described below in detail.

### 4.1. Electron Paramagnetic Resonance

Electron paramagnetic resonance (EPR) spectroscopy combined with site-directed spin labeling (SDSL) is a powerful biophysical technique for investigating the structural and dynamic properties of membrane proteins. However, the application of pulse EPR spectroscopy to determine intramolecular distances in spin-labeled membrane proteins in their native membrane-bound state is limited, mainly due to the inhomogeneous distribution of the proteins and the resulting intermolecular contributions. The use of lipodiscs as a novel membrane-mimetic system for EPR studies of membrane proteins appears to minimize this limitation, wherein the structure, dynamics, and function of the membrane protein under study are not impaired by the polymer encasement. EPR spectroscopy has already provided valuable information on the properties of membrane proteins enclosed in lipodiscs. EPR spectroscopy of the spin-labeled seven-transmembrane helix protein bacteriorhodopsin incorporated into lipodiscs provided protein dynamics similar to its state in the native membrane [63].

For the voltage-gated potassium channel KCNE1 having a single transmembrane helix, the EPR data showed improved quality for interspin distance measurements in SMALPs as compared to proteoliposomes [101,144]. Furthermore, the combination of continuous-wave (cw) and pulse EPR confirmed the stabilizing effect of SMALPs on the structure of the more complex membrane protein, the human KCNQ1 voltage-sensing domain with four transmembrane helices [145].

SMALPs were used for EPR studies of the conformational dynamics of sensory rhodopsin II [146]. The sensory rhodopsin II of *Natronomonas pharaonis* (*Np*SRII) is a membrane-embedded photoreceptor that mediates the photophobic response to potentially harmful blue light and plays a key role in negative phototaxis. It forms a transmembrane complex with its cognate protein transducer (*Np*HtrII). The cw and pulse EPR data showed that SMALPs generally maintain the dynamic features of the reconstituted *Np*SRII/*Np*HtrII complex. In addition, the transient cw EPR light–dark difference spectra revealed that SMALPs preserve the light-triggered conformational changes in both the encaged *Np*SRII and the *Np*SRII/*Np*HtrII complex, similar to liposomes and MSP-nanodiscs. However, the restricted spin-label side-chain mobility indicates that the protein is less flexible in SMALPs. *Np*SRII could also be solubilized into larger (up to 35 nm) lipodiscs using DIBMA [93]. The *Np*SRII photocycle in DIBMALPs depends on the lipid-to-protein ratio, underlining the importance of optimized preparation of protein-containing lipodiscs. The authors concluded that SMALPs and DIBMALPs could be suitable for the preparation of stable and functional membrane protein samples for (EPR) spectroscopic investigations of their conformation and dynamics, keeping in mind possible restrictions of conformational changes in the transmembrane region of the protein(s).

### 4.2. Nuclear Magnetic Resonance

Nuclear magnetic resonance is a powerful technique allowing one to characterize the structure and dynamics of membrane proteins solubilized in lipodiscs as well as their interactions with lipids and ligands [12]. The size of lipodiscs can be adjusted for both solution and solid-state NMR by varying the lipid:polymer ratio, as discussed in Section 2.2.1. Chemically modified large SMA-stabilized lipodiscs (“macrodiscs”) have been shown to align in a magnetic field, and a solid-state NMR spectrum of an associated protein has been obtained [23]. In [147], the coat protein of the Pf1 bacteriophage remained stable after solubilization by SMA, and the measured solid-state NMR signals were sharper than those in bicelles or peptide-based nanodiscs.

Daptomycin, a lipopeptide antibiotic, was studied by NMR in lipodiscs that contained DMPC and DMPG, stabilized by a styrene–maleic acid copolymer that was modified to reduce chelation by divalent ions. Daptomycin was found to form stable oligomers under physiologically relevant conditions [148].

High-resolution magic-angle-spinning solid-state NMR (MAS ssNMR) spectroscopy was used to characterize the integral homodimeric membrane protein CzcD from *Cupriavidus metallidurans* CH34, a zinc diffusion facilitator with a molecular weight of 34 kDa, solubilized in SMA-stabilized lipodiscs [149]. Additional examples of lipodisc applications in NMR spectroscopy are reviewed in [143]. However, while SMALPs have the potential to produce discs either small enough for solution-state NMR or big enough for solid-state NMR, their application in membrane protein structure determination by NMR has been limited to date [150].

### 4.3. Small-Angle Scattering

Small-angle X-ray and neutron scattering (SAXS and SANS) allow studies of supramolecular structure of soluble as well as membrane proteins (MPs) in solution. To use SAS methods for structural studies of MPs, the latter should be maintained in a native lipid environment or in membrane-mimicking systems. Lipid vesicles [151], MSP- or saposin-based phospholipid nanodiscs [152], lipodiscs based on SMA and other similar polymers, bicelles, and detergent micelles can be used as such.

In the case of MPs solubilized in a detergent, when an atomic model of the protein is available, the characteristic dimensions and shape of the detergent belt can be determined from SAXS data using the MEMPROT program [153]. For MPs reconstituted in nanodiscs, several approaches are currently available. One of them uses an atomic model of an MP and defines the positioning of the protein in the nanodisc that corresponds to the best approximation of SAXS data [154]. Another approach is realized in MPBuilder, a PyMOL plugin for the building and refinement of solubilized membrane proteins against small-angle X-ray scattering data [155]. MPBuilder constructs atomistic models of membrane-mimicking systems and covers the cases of MSP or Salipro nanodiscs as well as detergent belts and bicelles; however, it also needs an atomic model of a membrane protein as the input data. The third approach is realized in the Marbles program [156]; it predicts the shape of membrane proteins embedded into nanodiscs using a hybrid approach that accounts for nanodiscs’ contribution to the SAXS intensity through a semianalytical model, while the embedded membrane protein is treated as a set of beads, similarly to as in well-known ab initio methods. Today, Marbles is the only approach for ab initio shape determination of MPs reconstituted in nanodiscs, and it combines shape determination with a search for the position of the protein in the nanodisc.

It is important to note that the classical ab initio approaches such as those used in DAMMIF [157], which work well for soluble proteins, do not take into account the electron density inhomogeneities that are present in protein/lipid or protein/detergent complexes. Therefore, the direct application of these classical ab initio approaches to the SAXS data obtained for MPs may provide misleading results. For example, the presence of detergents or lipids bound to MPs results in the appearance of scattering intensity maxima at q ≈ 0.1–0.2 Å^−1^ (demonstrated for the sensory rhodopsin II/transducer complex from *Natronomonas pharaonis* [158,159]). In SMA-solubilized lipodiscs, the polymer may be located not only at the rim of the lipodisc, but also on its surface, affecting the scattering intensity [65]. In the case of SANS data obtained for MPs, inhomogeneities of scattering length density are not so crucial, and direct application of the classical ab initio approaches is possible in most cases.

Since solubilization using SMA and other similar polymers is a relatively new method, there is currently no universal approach for constructing models of proteins in lipodiscs. However, the two available SAXS/SANS studies of MPs reconstituted to SMA-based lipodiscs, which will be discussed below, show that the application of classical ab initio approaches directly to the SAXS data may give correct models, in contrast with the examples mentioned earlier.

The first published example of using SMALPs to study a membrane protein using SAXS is the work by Lee et al. [124]. In the study, *E. coli* membrane protein ZipA was isolated in a native lipid environment using 2:1 (S:M ratio) SMA. Standalone ZipA and ZipA in complex with the bacterial tubulin homolog FtsZ were investigated by cryo-electron microscopy and small-angle X-ray and neutron scattering. From the SAXS data, the main characteristics of the structures of the complexes (radius of gyration and maximum dimension) were obtained, and an ab initio model was built, according to which it was difficult to determine where the lipid disk was located. This observation can be explained by the hypothesis that lipid disks in the SMALP-ZipA samples could have a different size and shape compared to the case of empty SMA 3:1 lipodiscs. Experimental SAXS data shown in this work do not have the maxima of scattering intensity at q ≈ 0.1–0.2 Å^−1^, which is typical for SAXS data from MPs reconstituted in other membrane-mimicking systems. This work shows a good agreement between ab initio models obtained directly from SAXS and SANS data; it points to the idea that inhomogeneities of electron densities in MP/lipids/SMA complexes are not so crucial as in the cases of other membrane-mimicking systems mentioned above.

**Figure 4:**
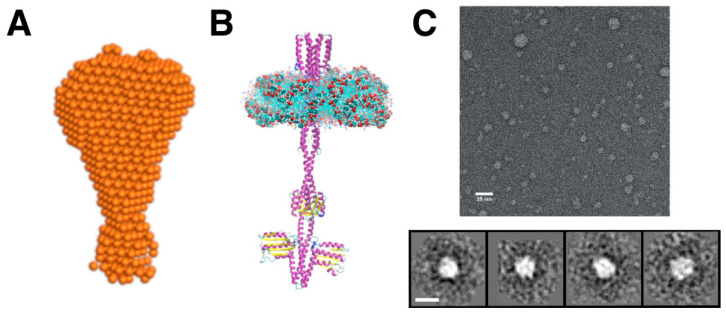
Structural characterization of proteins in lipodiscs. (**A**) Ab initio model of the full-length sensor histidine kinase *Ec*NarQ generated by DAMMIN; (**B**) atomistic model of the full-length *Ec*NarQ embedded in a SMALP; (**C**) TEM image of the SMA lipodiscs containing K_v_7.1 potassium channel along with the representative examples of two-dimensional class images. The scale bar for the classes corresponds to 10 nm.

The second example of using SMALPs for SAXS studies of membrane proteins was presented by Nazarenko et al. [160] (see Figure 4A,B). In this example, the combination of SAXS with size-exclusion chromatography (SEC) was used for structural characterization of the full-length nitrate/nitrite sensor histidine kinase NarQ [161] from *Escherichia coli* extracted from native *E. coli* membrane with SMA polymer. As in the previous example [124], SAXS curves do not demonstrate the maxima of scattering intensity at q ≈ 0.1–0.2 Å^−1^. Yet, the ab initio model generated by DAMMIN and the atomic model of the full-length NarQ dimer embedded in a SMALP (see Figure 4) shown in [160] can be considered as well matched to each other (taking into account the resolution of ~60 Å for the dataset used).

Although published data on the studies of membrane proteins in lipodiscs using SAS are limited, this technique can certainly provide valuable complementary information for structural studies. Existing examples of applying SAS for MPs reconstituted to SMALPs show that classical ab initio approaches applied directly to the SAXS data may give correct models, in contrast to other membrane-mimicking systems such as nanodiscs and micelles. For the latter, analysis of SAXS data may require taking into account more parameters than in the analysis of soluble proteins. However, more examples of SAXS studies of MPs in SMALPs and other kinds of lipodiscs are needed to validate this idea.

### 4.4. X-ray Crystallography

X-ray crystallography remains the method of choice when high-resolution structural information is desirable. In some cases, MPs solubilized in detergents can be crystallized similarly to soluble proteins, using the in surfo approaches, whereas in others, various lipidic mesophases and membrane-mimicking systems are employed in so-called in meso crystallization methods [162,163]. The former are favored for larger proteins with extended solvent-exposed surfaces, whereas the latter are well suited for crystallization of smaller proteins such as microbial rhodopsins or G-protein-coupled receptors.

Successful direct crystallization of a SMALP-embedded membrane protein has not been reported yet. However, SMA could be used to solubilize bacteriorhodopsin from *Haloquadratum walsbyi* and transfer it to monoolein lipidic cubic phase [164] for subsequent crystallization and determination of structure at the resolution of 2.0 Å [165]. Similarly, amphipols [166] and nanodiscs [167] were used to obtain structures of bacteriorhodopsin by in meso approaches at the resolutions of 2.0 and 1.9 Å, respectively, although they could not be used for solubilization. Thus, it is likely that amphiphilic copolymers and solubilizing agents in general are well suited for transferring MPs to lipidic mesophases and do not significantly interfere with the in meso crystallization process.

### 4.5. Electron Microscopy

The recent advances in the cryo-EM technique have revolutionized the structural biology of membrane proteins, allowing high-resolution structures of such proteins to be obtained only using microgram protein quantities and without the need for ordered crystals and long data collection times, in contrast to X-ray crystallography and NMR, respectively. With the break of the atomic resolution barrier [168] and the opportunity to capture different biologically relevant conformations of proteins by the state-of-the-art cryo-EM approaches [169,170], it is becoming important as never before to guarantee the presence of a native lipid environment in protein samples. Lipodiscs provide such an opportunity and therefore represent an advantageous platform for structural studies of membrane proteins.

Lipodiscs have been shown to be a valid solubilization platform for negative stain EM and low-resolution structure reconstruction (see Figure 4C) [129] despite the prevalence of specific orientations of the protein-loaded SMALPs most commonly observed in the top views [171]. Although this method offers only modest resolution (15–20 Å), its undeniable advantage is that the structures can be obtained in a short time frame of several days [172].

The first sub-nanometer cryo-EM structure of the *E. coli* multidrug efflux transporter AcrB solubilized in SMALP resulted in the density map consistent with high-resolution crystal structures and other EM-derived maps [173]. This study as well as another cryo-EM structure of the SMALP-embedded alternative complex III in a supercomplex with cytochrome oxidase [174] clearly proved the usefulness of SMA in cryo-EM studies. These pioneering studies were followed by a number of other successful examples, including the human TRPM4 ion channel [30], the human glycine receptor [131], the potassium importer KimA from *Bacillus subtilis* [125], and chicken ASIC1 ion channel [175] with the resolution ranging from 2.8 to 18 Å and the molecular mass of protein(s) up to 0.5 MDa. Although so far SMA remains the most commonly used lipodisc-forming polymer in cryo-EM studies, AASTY and DIBMA have proven to be suitable alternatives and have recently allowed researchers to determine the structures of the human TRPM4 ion channel [30] and bacterial MscS-like channel YnaI [176] at the resolutions of 18 and 3.0 Å, respectively. Importantly, in the latter study, the authors were able to determine both closed-like and open-like conformations of the channel.

## 5. Further Investigations, Perspectives and Conclusions

During recent years, a large body of results regarding lipodisc formation, structure, and dynamics, as well as the applicability of lipodiscs to various problems of structural biology, has been aggregated. It has allowed researchers to propose novel types of polymers with a wider range of conditions suitable for protein solubilization, and better suited for specific tasks, as well as to develop efficient protocols for analyzing the structure and dynamics of membrane proteins reconstituted in lipodiscs using different biophysical techniques. We expect that the repertoire of lipodisc-forming polymers will continue to grow, providing new versatile tools to deal with membrane proteins and their complexes, including extra-large ones currently remaining unreachable for reconstitution in lipodiscs.

One of the growing areas for applications of lipodiscs is the detailed analysis of specific annular lipids bound to membrane proteins. Since SMA and related copolymers do not preferentially solubilize any specific types of lipids, they appear as an ideal tool for unbiased analysis of lipid content in membrane:protein assemblies (termed “memteins” in [12]) using both structural approaches and LC-MS/MS methods [13]. Importantly, many pharmaceutically relevant targets (e.g., GPCRs [177]) are sensitive to their native lipid environment [178], and their solubilization in lipodiscs can be beneficial for the development of an accurate drug screening platform as well as for the fundamental understanding of how lipids modulate protein dynamics [143].

Another promising application of lipodiscs is obtaining the structures of membrane proteins from the single particles using X-FEL crystallography. The successful determination of the structure of single minivirus particles on the AMO instrument in the LCLS [179] supports the idea of using nanolipoprotein particles for such experiments. In a recent study [180], the general feasibility of the structure characterization by means of X-FEL crystallography of nanolipoprotein particles (NLPs) was demonstrated, and novel serial femtosecond crystallography (SFX) methods were developed based on the NLP membrane protein encapsulation. Such investigations can facilitate understanding the specific role of protein/lipid complexes in lipid binding and particle maturation dynamics. Analogously, lipodiscs can be used for the SFX experiments.

## Data Availability

The data presented in this study are available on request from the corresponding author.

## Abbreviations

MPs	membrane proteins
SMA	copolymer of polystyrene and maleic anhydride
XFEL	X-ray free-electron laser
SMAD	styrene–maleic anhydride copolymer derivative
DIBMA	diisobutylene–maleic acid copolymer
DLS	dynamic light scattering
SEC	size-exclusion chromatography
EPR	electron paramagnetic resonance
SAXS/SANS	small angle x-ray/neutron scattering
SMALP	styrene–maleic anhydride copolymer lipoprotein particle
MSP	membrane scaffolding protein
MACPs	maleic acid-containing alternating copolymers
LC	liquid chromatography
MS	mass spectrometry
